# Differential Impact of SARS-CoV-2 Isolates, Namely, the Wuhan Strain, Delta, and Omicron Variants on Erythropoiesis

**DOI:** 10.1128/spectrum.01730-22

**Published:** 2022-08-09

**Authors:** Suguru Saito, Shima Shahbaz, Wendy Sligl, Mohammed Osman, D. Lorne Tyrrell, Shokrollah Elahi

**Affiliations:** a School of Dentistry, Division of Foundational Sciences, University of Alberta, Edmonton, Alberta, Canada; b Department of Critical Care Medicine, University of Alberta, Edmonton, Alberta, Canada; c Department of Medicine, Division of Infectious Diseases, University of Alberta, Edmonton, Alberta, Canada; d Department of Medicine, Division of Rheumatology, University of Alberta, Edmonton, Alberta, Canada; e Department of Medical Microbiology and Immunology, University of Alberta, Edmonton, Alberta, Canada; f Li Ka Shing Institute of Virology, University of Alberta, Edmonton, Alberta, Canada; g Department of Oncology, Faculty of Medicine and Dentistry, University of Alberta, Edmonton, Alberta, Canada; h Women and Children Health Research Institute, University of Alberta, Edmonton, Alberta, Canada; Indian Institute of Science Bangalore

**Keywords:** Delta, erythropoiesis, Omicron, SARS-CoV-2, SARS-CoV-2 variants, Wuhan

## Abstract

SARS-CoV-2 variants exhibit different viral transmissibility and disease severity. However, their impact on erythropoiesis has not been investigated. Here, we show SARS-CoV-2 variants differentially affect erythropoiesis. This is illustrated by the abundance of CD71^+^ erythroid cells (CECs) in the blood circulation of COVID-19 patients infected with the original Wuhan strain followed by the Delta and Omicron variants. We observed the CD45^+^CECs are the dominant subpopulation of CECs expressing the receptor, ACE2, and coreceptor, TMPRSS2, and thus, can be targeted by SARS-CoV-2. Also, we found CECs exhibit immunosuppressive properties, specifically CD45^+^CECs are the dominant immunosuppressive cells and via reactive oxygen species (ROS) and arginase I expression can impair CD8^+^ T cell functions. In agreement, we observed CECs suppress CD8^+^ T cell effector (e.g., Granzyme B expression and degranulation capacity [CD107]), which was partially but significantly reversed with l-arginine supplementation. In light of the enriched frequency of CECs, in particular, CD45^+^CECs in patients infected with the original (Wuhan) strain, we believe this strain has a more prominent impact on hematopoiesis compared with the Delta and Omicron variants. Therefore, our study provides an important insight into the differential impact of SARS-CoV-2 variants on erythropoiesis in COVID-19 patients.

**IMPORTANCE** Silent hypoxia has been the hallmark of SARS-CoV-2 infection. Red blood cells (RBCs) work as gas cargo delivering oxygen to different tissues. However, their immature counterparts reside in the bone marrow and normally absent in the blood circulation. We show SARS-CoV-2 infection is associated with the emergence of immature RBCs so called CD71^+^ erythroid cells (CECs) in the blood. In particular, we found these cells were more prevalent in the blood of those infected with the SARS-CoV-2 original strain (Wuhan) followed by the Delta and Omicron variants. This suggests SARS-CoV-2 directly or indirectly impacts RBC production. In agreement, we observed immature RBCs express the receptor (ACE2) and coreceptor (TMPRSS2) for SARS-CoV-2. CECs suppress T cells functions (e.g., Granzyme B and degranulation capacity) *in vitro*. Therefore, our study provides a novel insight into the differential impact of SARS-CoV-2 variants on erythropoiesis and subsequently the hypoxia commonly observed in COVID-19 patients.

## INTRODUCTION

There have been multiple SARS-CoV-2 variants identified by genomic sequencing. The original (Wuhan) strain subsequently evolved into Delta and Omicron variants, both of which have spread globally and caused significant morbidity and mortality. SARS-CoV-2 infection manifests as corona virus disease (COVID-19), including acute respiratory distress syndrome (ARDS) in a subgroup of patients—a clinical phenomenon associated with the development of bilateral lung infiltrates and hypoxemia (1). However, the impact of different viral variants on erythropoiesis has not been well-established. It is suggested that SARS-CoV-2 may inhibit heme metabolism and induce hemoglobin denaturation (2), compromising the oxygen-carrying capacity of red blood cells (RBCs). Also, structural protein damage and modifications in RBC membrane lipids have been observed in COVID-19 patients (3). Moreover, unusual RBC morphological abnormalities such as elevated RBC distribution width (RDW), a standard component of RBC quality assessment, has been reported in COVID-19 patients (4). Erythroid progenitors/precursors are defined as CD71^+^ erythroid cells (CECs) coexpressing CD71 (the transferrin receptor) and CD235a (glycophorin A, erythroid lineage marker) in humans (5–7). Notably, we and others reported the SARS-CoV-2’s Wuhan strain directly invades erythroid progenitors (8, 9). Hence, elimination of erythroid progenitors by SARS-CoV-2 may cause stress erythropoiesis, a compensatory mechanism to meet the oxygen supply, resulting in the premature egress of erythroid progenitors and precursors from the bone marrow to the blood (10). In line with these observations, multiple studies have emerged to imply COVID-19 disease is associated with RBC abnormalities. For example, dysregulated iron homeostasis has been observed in SARS-CoV-2 infected individuals (11).

In light of the above, we aimed to investigate and compare the impact of different SARS-CoV-2 viral strains namely, the Wuhan strain, as well as Delta and Omicron variants on erythropoiesis by characterizing the frequency of CECs in the fresh blood of ICU-admitted COVID-19 patients.

## RESULTS AND DISCUSSION

**FIG 1:**
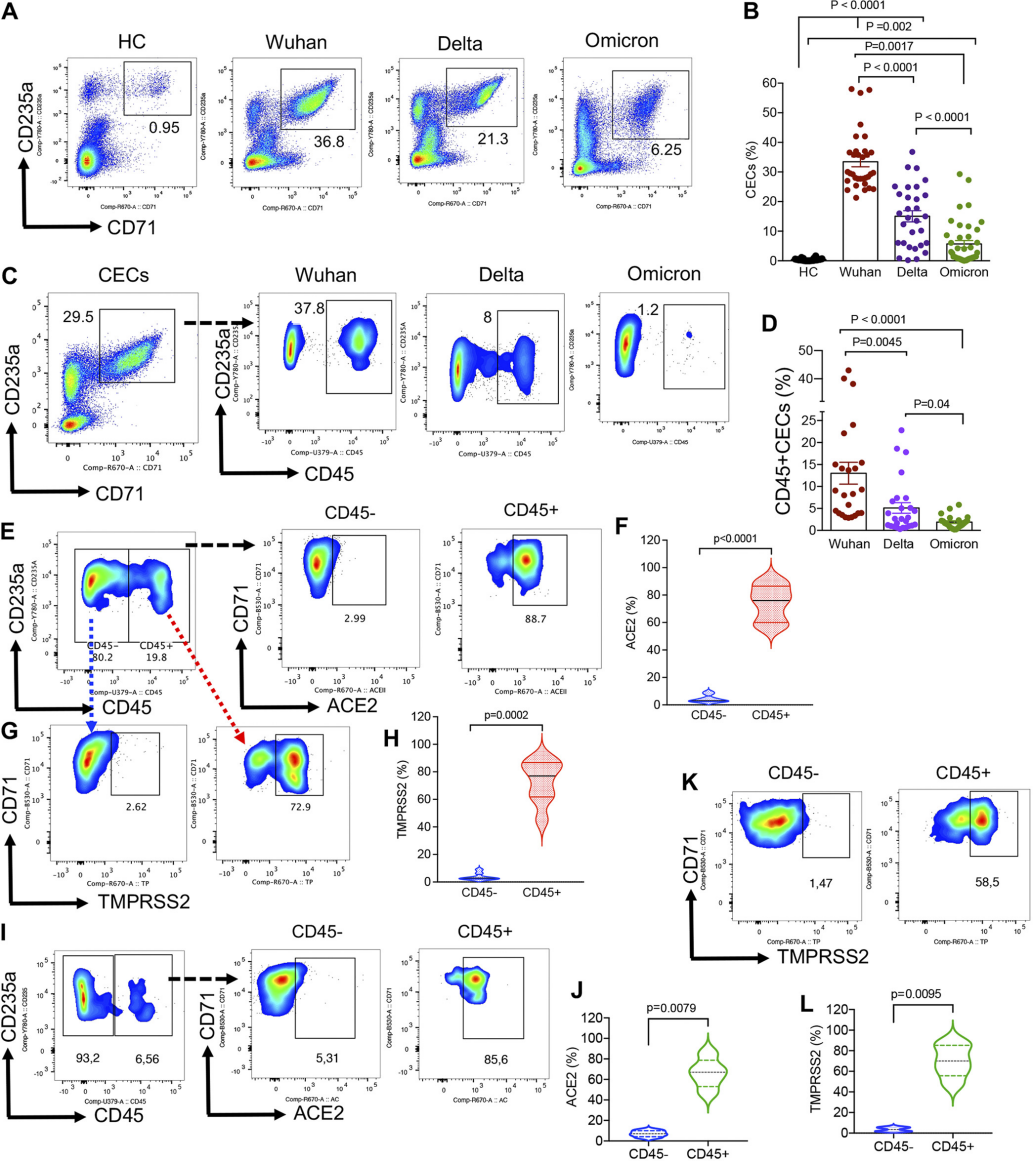
The frequency of CD71^+^ erythroid cells (CECs) resemble differential effects of SARS-CoV-2 variants on hematopoiesis. (A) Representative flow cytometry plots, and (B) cumulative data of the frequency of CECs in the peripheral blood mononuclear cells (PBMCs) of healthy controls (HC) and infected individuals with the Wuhan strain, delta, and Omicron variants. (C) Representative flow cytometry plots, and (D) cumulative data of the proportion of CD45^−^ and CD45^+^CECs in PBMCs of infected individuals with different SARS-CoV-2 isolated. (E) Representative flow cytometry plots, and (F) cumulative data of the percentages of ACE2 expressing CECs among CD45^−^ and CD45^+^ subpopulations of CECs in patients infected with the Wuhan strain. (G) Representative flow cytometry plots, and (H) cumulative data of the percentages of TMPRSS2 expressing CECs among CD45^−^ and CD45^+^ subpopulations of CECs in patients infected with the Wuhan strain. (I) Representative flow cytometry plots, and (J) cumulative results of the percentages of ACE2 expressing CECs among CD45^−^ and CD45^+^ subpopulations of CECs in patients infected with the Omicron variant. (K) Representative flow cytometry plots, and (L) cumulative results of the percentages of TMPRSS2 expressing CECs among CD45^−^ and CD45^+^ subpopulations of CECs in patients infected with the Omicron variant. Each dot represents data from an individual patient or study subject.

In our previous study (9), we observed SARS-CoV-2 infection had the most prominent effects on hematopoiesis in patients admitted to the ICU. Therefore, in this report, we focused on this group and analyzed the frequency of CECs in COVID-19 patients infected with different SARS-CoV-2 strains. Interestingly, we observed the frequency of CECs was significantly lower in the peripheral blood of SARS-CoV-2 Delta variant-infected individuals compared with those infected with the Wuhan strain (Fig. 1A and B). However, the frequency of CECs was significantly higher in infected individuals with the Delta variant compared with the Omicron variant (Fig. 1A and 1B). Of note, those infected with the Omicron variant had a significantly smaller proportion of CECs in their blood compared with those infected with either the Wuhan or Delta isolates (Fig. 1A and B). Nevertheless, the frequency of CECs in healthy controls (HCs) was very low or negligible as reported elsewhere (12) (Fig. 1A and B). CECs can be divided based on the surface expression of CD45 into CD45^+^ or CD45^−^CECs. CD45^+^CECs are considered early erythroid progenitors but CD45^−^CECs as RBC precursors (9, 10). We found that the proportion of CD45^+^CECs was significantly greater in patients infected with the Wuhan strain compared to the Delta and the Omicron variants (Fig. 1C and D).

Considering erythroid progenitors possess nuclei and can support viral replication (8, 9), we found the SARS-CoV-2 receptor, ACE2, was prominently expressed in CD45^+^CECs compared with their older siblings in patients infected with the Wuhan strain (Fig. 1E and F). Similarly, we observed CD45^+^CECs were the dominant cells expressing the SARS-CoV-2 coreceptor (TMPRSS2) in these patients (Fig. 1G and H). Moreover, we analyzed the expression of ACE2 and TMPRSS2 in CECs from patients infected with the Omicron variant, which revealed CD45^+^CECs as the major cells expressing SARS-CoV-2 receptor and coreceptor (Fig. 1I to L). Overall, CD45^+^CECs are the dominant population of cells which are affected by SARS-CoV-2 infection. As such, a higher proportion of CD45^+^CECs in infected individuals with the Wuhan strain compared with Delta and Omicron variants implies CECs in these patients are more prone to infection and subsequently the Wuhan strain may have a more pronounced impact on erythropoiesis compared with Delta and Omicron variants (Fig. S1).

**FIG 2:**
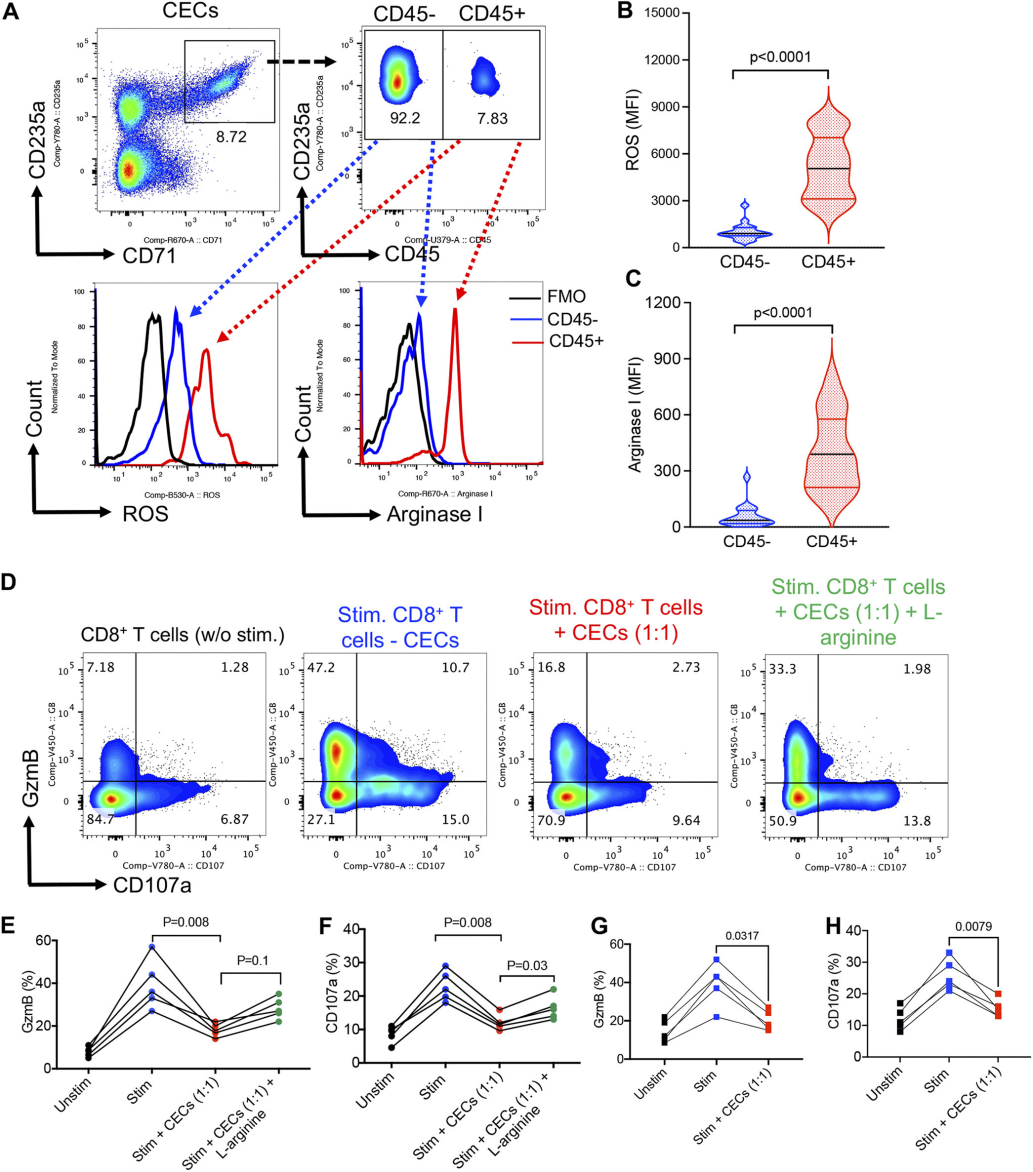
CD45^+^CECs in COVID-19 patients are more potent. (A) Representative flow cytometry plots of the gating strategy for CD45^−^ and CD45^+^CECs, and the expression of ROS and arginase I in CD45^−^ versus CD45^+^CECs. (B) Cumulative data of the intensity of ROS expression measured by mean fluorescence intensity (MFI) in CD45^−^ versus CD45^+^CECs. (C) Cumulative data of the intensity (MFI) of arginase I expression in CD45^−^ versus CD45^+^CECs. (D) Representative flow cytometry plots, and (E) cumulative data showing % CD8^+^ T cells expressing GzmB, or (F) CD107a without stimulation (unstim) or stimulated (stim) with anti-CD3/CD28 antibodies in the absence of CECs (−CECs) or presence (+CECs) without and with l-arginine supplementation (2 mM). (G) Cumulative data showing % CD8^+^ T cells expressing GzmB, or (H) CD107a without stimulation (Unstim) or stimulated (stim) with anti-CD3/CD28 antibodies in the absence of CECs (−CECs) or presence (+CECs). Each dot represents data from a human subject.

Due to their immunosuppressive properties, the pathological abundance of CECs in COVID-19 patients can have immunological consequences (5, 6, 10). We found CD45^+^CECs express significantly higher amounts of ROS (Fig. 2A and B) and arginase I (Fig. 2A and C) compared with their CD45^−^ counterparts (13, 14). This is similar to what has been reported in CD45^+^CECs in newborns (15), HIV (16), and cancer patients (14). In turn, CECs isolated from infected patients with SARS-CoV-2 Wuhan strain significantly suppressed granzym B (GzmB) expression and degranulation capacity (CD107a) of CD8^+^ T cells, which was partially but significantly reversed in the presence of l-arginine supplementation (Fig. 2D to F) as reported elsewhere (7, 17). Similar observations were made for the suppression of GzmB/CD107a expression in CD8^+^ T cells by SARS-CoV-2 delta variant (Fig. 2G and H). Although we were unable to examine CECs from infected patients with SARS-CoV-2 Omicron variant due to their lower frequency, we speculate CECs from these patients exhibit similar immunosuppressive function. These observations demonstrate the immunosuppressive properties of CECs in COVID-19 patients, potentially resulting in the impairment of CD8^+^ T cells effector functions.

The present study is the first to describe how SARS-CoV2 variants influence erythropoiesis in patients admitted to the ICU. We identified the significant expansion of CECs in the peripheral blood of COVID-19 patients, in particular, in those infected with the Wuhan strain. The expression of ACE2 and TMPRSS2 by CD45^+^CECs RBC may imply direct invasions of erythroid progenitors by SARS-CoV-2, which may explain one potential mechanism for the observed hypoxia in COVID-19 patients. As such, a higher proportion of CECs in infected patients with the Wuhan strain might be the result of a greater stress hematopoiesis in these infected individuals. The disease severity/cytokine storm could fuel stress hematopoiesis and the expansion of CECs in the peripheral blood. Subsequently, CECs become the target of SARS-CoV-2 resulting in their depletion. In line with our findings, anemia has been considered an independent risk factor for severe COVID-19 disease (18, 19). We hypothesize this phenomenon might be in part due to the elimination of infected/damaged CECs by lysis or/and phagocytosis. On the other hand, CECs due to their immunosuppressive nature may compromise adaptive immune response against SARS-CoV-2 infection.

## MATERIALS AND METHODS

### Ethics statement.

The Human Research Ethics Board (HREB) at the University of Alberta approved the study (Pro00099502) in hospitalized patients. Similarly, ethics approval was obtained for blood collection from healthy controls (Pro00063463, *n* = 20, age 50±10).

### Sample collection and processing.

Blood samples were collected from 103 ICU-admitted COVID-19 patients in Edmonton, Alberta: Wuhan (*n* = 33, age 56 ± 11), Delta (*n* = 30, age 55 ± 13), and Omicron (*n* = 40, age 52 ± 15) strains. All COVID-19 patients were SARS-CoV-2 positive by RT-PCR assay specific for viral RNA-dependent RNA polymerase and envelope transcripts detected using nasopharyngeal swab or endotracheal aspirates. Fresh peripheral blood mononuclear cells (PBMCs) were isolated over Ficoll (GE) gradients. CEC isolation was performed according to our previous reports (7, 12, 20). To determine their functionality, CECs were isolated from the PBMCs with >95% purity according to our previous reports (21), then cocultured with CECs-depleted autologous PBMCs.

### Flow cytometry antibodies and flow cytometry.

Fluorophore antibodies with specificity to human cell surface antigens were purchased mainly from BD Biosciences or Thermo Fisher Scientific. Specifically, the following antibodies were used: anti-CD3 (HIT3a), anti-CD4 (RPA-T4), anti-CD8 (RPA-T8), anti-CD45 (H-130 or 2D1), anti-CD107a (H4A3), anti–Granzyme B (GzmB; GB11), anti-CD71 (MA712), anti-CD235A (HIR2), and arginase I (IC5868N/R&D). In addition, anti-ACE2 (535919) from R&D, anti-TMPRSS2 (EPR3862) from abcam, and DCFDA (Sigma) were used for staining. Besides, live/dead fixable dead cell stains (Thermo Fisher) were used to exclude dead cells in flow cytometry. Paraformaldehyde fixed cells were acquired by flow cytometry using an LSRFORTESSA flow cytometer (BD) and analyzed with FlowJo software.

### Coculture and stimulation.

For coculture, a fixed number (1 × 10^6^) of PBMCs were seeded into 96-well round-bottom plates individually or together with autologous CECs at 1:1 ratio. Brefeldin A (1 μg/mL) was added at the same time and the cells were stimulated with anti-CD3 (3 μg/mL) and anti-CD28 (1 μg/mL) antibodies for 6 h.

### Statistical analysis.

Statistical comparisons between various groups were performed by using *t* test and Mann-Whitney tests (as appropriate). Results are expressed as mean ± SEM. *P* value < 0.05 was considered statistically significant.
