# Supplementary material for: Differential Impact of SARS-CoV-2 Isolates, Namely, the Wuhan Strain, Delta, and Omicron Variants on Erythropoiesis
Source: Microbiol Spectr. 2022 Aug 9;10(4):e01730-22. doi: 10.1128/spectrum.01730-22 (PMC9430111; doi:10.1128/spectrum.01730-22)
Supplement: Supplemental file 1 — Supplemental material. Download spectrum.01730-22-s0001.pdf, PDF file, 0.3 MB [file spectrum.01730-22-s0001.pdf]

Supplemental Figure 1

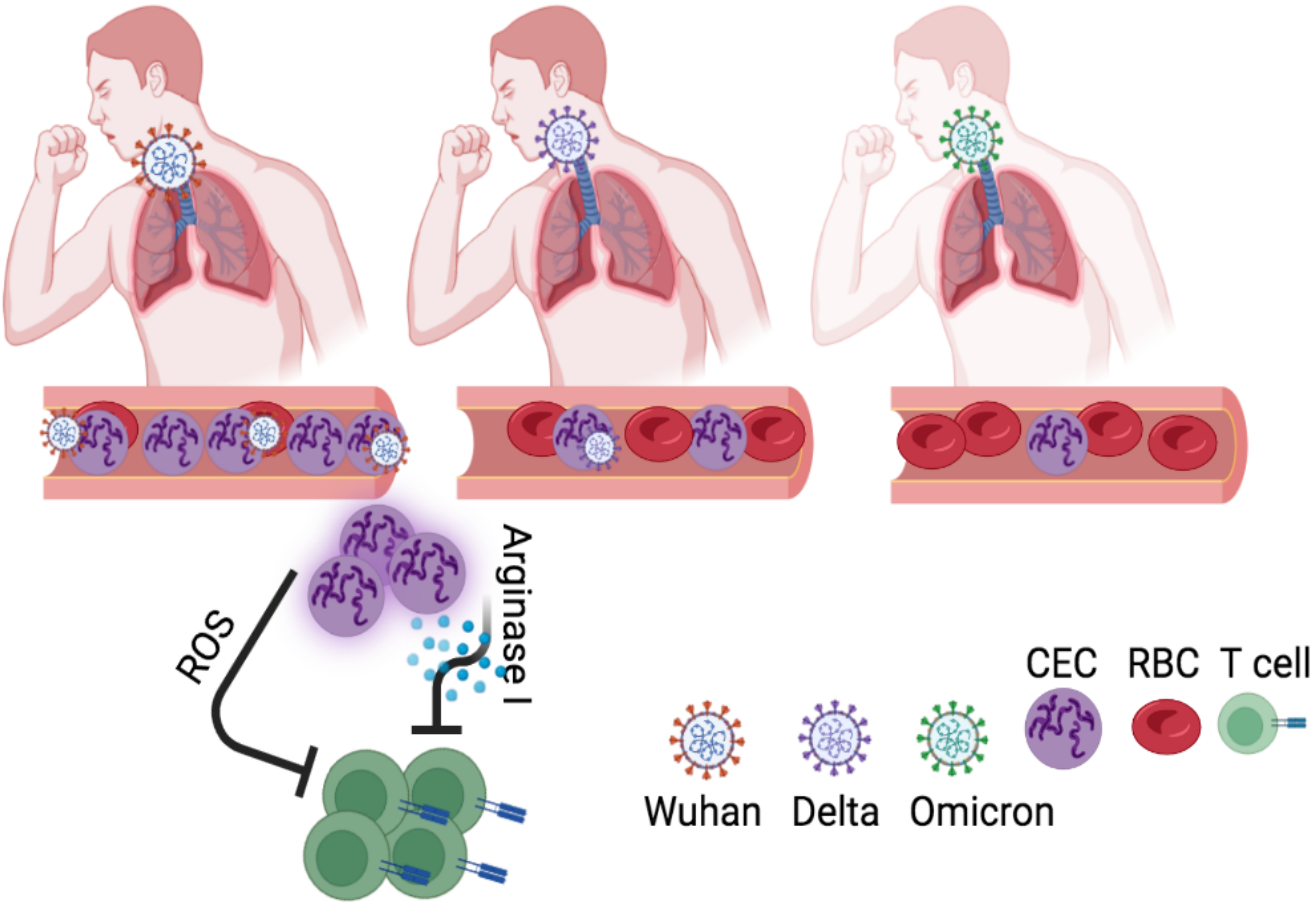

This illustrates the higher frequency of CECs in the blood circulation of patients infected with the Wuhan strain followed by Delta and Omicron variants. Subsequently, CECs via ROS and arginase I may suppress CD8 T cell effector functions.
